# MHEALTH PROTOTYPE AND PILOT PROTOCOL TO ENHANCE SOCIAL SUPPORT FOR PERSONS LIVING WITH DEMENTIA

**DOI:** 10.1093/geroni/igz038.279

**Published:** 2019-11-08

**Authors:** Kirsten Corazzini, Donald (chip) Bailey, Kayla Wright-Freeman, Eleanor McConnell

**Affiliations:** 1 Duke University, Durham, North Carolina, United States; 2 Duke, Hillsborough, North Carolina, United States

## Abstract

An emerging component of mHealth is the use of tailored mobile applications (app) to facilitate self-management of chronic illnesses, including the mapping of social networks to assist adults living with chronic illnesses to help them be able to identify sources of social support. The purpose of this study is to describe a prototype app to support persons living with dementia (PLWD) in the community and their informal caregivers to map social networks and identify sources of emotional, instrumental, informational, and appraisal of social support. Adapting the Network Canvas open-source software and drawing upon a previously-developed mobile application for adults to self-manage chronic illnesses, we share the key specifications, including health care provider output, preliminary end user feedback, and the pilot protocol designed to test the feasibility. Findings illustrate the importance of leveraging social network data in novel ways to enhance self-management and well-being among PLWD and their caregivers

